# Synthesis of new series of quinoline derivatives with insecticidal effects on larval vectors of malaria and dengue diseases

**DOI:** 10.1038/s41598-022-08397-5

**Published:** 2022-03-19

**Authors:** Kadarkarai Murugan, Chellasamy Panneerselvam, Jayapal Subramaniam, Manickam Paulpandi, Rajapandian Rajaganesh, Murugan Vasanthakumaran, Jagannathan Madhavan, S. Syed Shafi, Mathath Roni, Johan S. Portilla-Pulido, Stelia C. Mendez, Jonny E. Duque, Lan Wang, Al Thabiani Aziz, Balamurugan Chandramohan, Devakumar Dinesh, Shanmughavel Piramanayagam, Jiang-Shiou Hwang

**Affiliations:** 1 University of Science & Technology, Techno City, Kiling Road, Baridua, Meghalaya 793 101 India; 2 Division of Entomology, Department of Zoology, School of Life Sciences, Bharathiar University, Coimbatore, Tamil Nadu 641046 India; 3 Department of Biology, Faculty of Science, University of Tabuk, Tabuk, 71491 Saudi Arabia; 4 Department of Zoology, Kongunadu Arts and Science College, Coimbatore, 641029 India; 5 Department of Chemistry, Thiruvalluvar University, Serkadu, Vellore, 632 115 India; 6 Grupo de Investigación en Bioquímica y Microbiología (GIBIM). Escuela de Química, Universidad Industrial de Santander, A.A. 678, Bucaramanga, Colombia; 7 Centro de Investigaciones en Enfermedades Tropicales-CINTROP, Facultad de Salud, Escuela de Medicina, Departamento de Ciencias Básicas, Universidad Industrial de Santander, Guatiguará Technology and Research Park, Km 2 Vía El Refugio, Piedecuesta, Santander Colombia; 8 School of Life Science, Shanxi University, Taiyuan, 030006 Shanxi China; 9 Computational Biology Lab, Department of Bioinformatics, Bharathiar University, Coimbatore, Tamil Nadu 641046 India; 10 Institute of Marine Biology, National Taiwan Ocean University, Keelung, 20224 Taiwan; 11 Center of Excellence for Ocean Engineering, National Taiwan Ocean University, Keelung, 20224 Taiwan; 12 Center of Excellence for the Oceans, National Taiwan Ocean University, Keelung, 20224 Taiwan

**Keywords:** Biochemistry, Biological techniques, Biotechnology

## Abstract

Mosquito borne diseases are on the rise because of their fast spread worldwide and the lack of effective treatments. Here we are focusing on the development of a novel anti-malarial and virucidal agent with biocidal effects also on its vectors. We have synthesized a new quinoline (4,7-dichloroquinoline) derivative which showed significant larvicidal and pupicidal properties against a malarial and a dengue vector and a lethal toxicity ranging from 4.408 µM/mL (first instar larvae) to 7.958 µM/mL (pupal populations) for *Anopheles stephensi* and 5.016 µM/mL (larva 1) to 10.669 µM/mL (pupae) for *Aedes aegypti*. *In-vitro* antiplasmodial efficacy of 4,7-dichloroquinoline revealed a significant growth inhibition of both sensitive strains of *Plasmodium falciparum* with IC_50_ values of 6.7 nM (CQ-s) and 8.5 nM (CQ-r). Chloroquine IC_50_ values, as control, were 23 nM (CQ-s), and 27.5 nM (CQ-r). In vivo antiplasmodial studies with *P. falciparum* infected mice showed an effect of 4,7-dichloroquinoline compared to chloroquine. The quinoline compound showed significant activity against the viral pathogen serotype 2 (DENV-2). In vitro conditions and the purified quinoline exhibited insignificant toxicity on the host system up to 100 µM/mL. Overall, 4,7-dichloroquinoline could provide a good anti-vectorial and anti-malarial agent.

## Introduction

Vector-borne maladies are providing a serious threat to the well-being and public health around the world. Malaria, or dschungle fever, a tropical parasitic illness caused by the eukaryotic protest *Plasmodium* spp., provides one of the most significant infections on the planet^1^. An assessed 3.3 billion of the world human population lives in areas with risk of Malaria infection^2^ is contaminated with its mosquito vector *Anopheles* spp. Despite being preventable and treatable, malaria continues to provide severe effects on public health and livelihood in the tropical world^3,4^.

According to the World Health Organization^5^, almost two million people in the Americas suffered from dengue virus infection in 2019, and more recent data showed that four billion people suffer from dengue and related viruses such as Zika and Chikungunya in 128 countries worldwide^6^.

Quinoline provides a well studied compound and shows potential biological activities against vector borne diseases^7,8^. Quinoline provided the first anti-malarial medicine. It is a special kind of alkaloid originating from the herbal tree Cinchona^9^. By altering the places of the chemical aldehyde groups, quinoline increases its pesticidal properties^10^. Chloroquine provides well known clinical uses because of its viability and its generally safe application^11^. Attributable to huge natural bioactivities, quinoline compounds have attracted increasingly more consideration in combinatorial and bioactivity research^12,13^.

**Figure 1:**
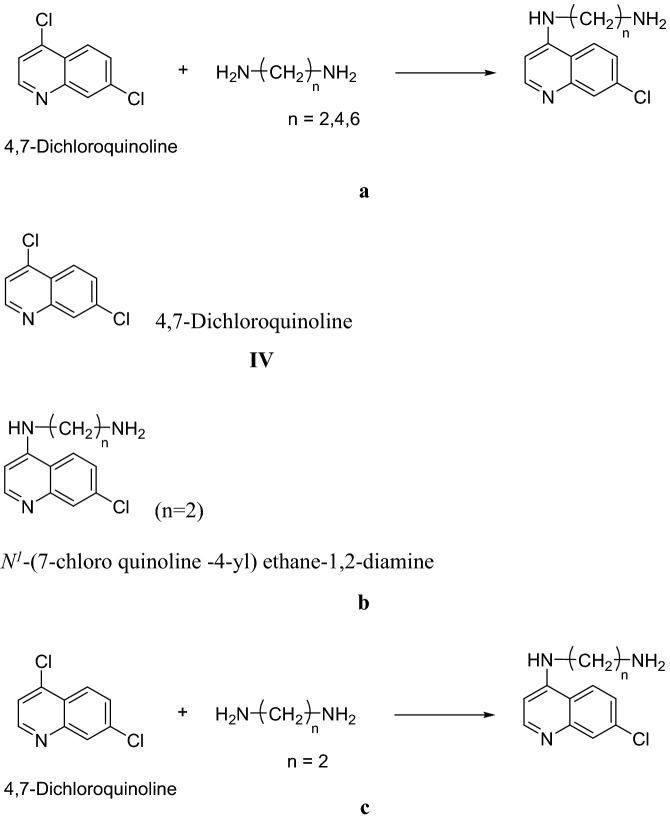
(**a**) 4,7-Dichloroquinoline design inspired by the natural molecule, chloroquine. (**b**) 4,7-Dichloroquinoline design inspired by the natural molecule, chloroquine. (**c**) 4,7-Dichloroquinoline design inspired by the natural molecule, chloroquine.

Quinoline subsidiaries have widespread biopharmaceutical applications^14^ (Fig. 1). Analysts have just decided numerous helpful bioactivities of quinoline subordinates, including among others mitigative effects, against bacteria^15,16^, hostility to viruses^17^ and cell reinforcement^18^. Therapeutic scientists incorporated an assortment of quinoline compounds with various natural compounds by introducing different dynamic gatherings to the quinoline moiety, utilizing engineering techniques and the possible utilization of quinoline subsidiaries in different fields of science, pesticide development and biomedicine^19–21^. Since 2011, several quinoline compounds have shown Epidermal Growth Factor Receptor (EGFR) inhibition^22^.

Among the heterocyclic compounds, 4,7-dichloroquinoline is a hydroxychloroquine intermediate for the treatment of different types of malaria^23^. Recently, numerous examinations are carried out with hydroxychloroquine for the therapeutic/forestall of pandemic COVID 19^24^. Likewise, the above said molecule was significant for the understanding of life performances^25,26^.

We synthesized a *N*^1^-(7-chloroquinoline-4-yl) ethane-1,2-diamine derivative by the method of Shafi et al.^8^ against *A. stephensi* providing no harmful impacts on the environment as well on non-target organisms (see Nyberg et al.^27^). As the plasmodium parasite becomes more resistant to quinoline based anti-malarial drugs, it becomes even more important to design a potent anti-malarial molecule^28,29^.

Hence, finding new compounds to treat malaria is urgently needed for the treatment of dangerous mosquito borne diseases^30,31^. This work provides a general overview of quinoline advantages for the discovery of more efficient compounds^32,33^. In continuation of the study for the preparation of a 4-diamine substituted-7-dichloroquinoline compounds against vector borne diseases^34^ we report herein the anti-malarial and anti-dengue potential of a novel quinoline compound.

The quinoline skeleton is utilized for some important engineered agrochemicals and to plan manufactured mixtures providing several pharmacological effects. Quinoline and its related compounds belongs to a significant class of antimalarial sedates that affect the parasite’s hemoglobin breakdown pathway. Earlier studies reported that for some time this compound was utilizing quinoline to battle malaria^35^. Along these lines, it is significant to re-look into the antimalarial movement of existing quinoline libraries or blend some unique quinoline subsidiaries with improved action. A methodical and broad investigation is needed to find a compelling antimalarial compound structure 4-aminoquinoline based framework^36^. In the present research, we have orchestrated several analogs of 4,7-dichloroquinoline and screened against jungle fever parasites, dengue (DENV-2) and their respective mosquito vectors. Also, we reported the synthesis of N2-2-((7-chloroquinolin-4-yl) amino) ethyl)-N4, N6-bis(4-nitrophenyl)-1,3,5-triazine-2,4,6-triamine. Whose synthesis have been planned for the bi-substituted cyanuric chloride using p-nitroaniline incorporated N1-(7-chloroquinoline–4–yl) ethane-1,2–diamine. Synthesized molecules can be analyzed by IR, ^1^HNMR, ^13^C, mass and elemental analysis to characterize their molecular structure. This is a new compound that is easily synthesized by substituting cyanuric chloride to provide s-triazine derivatives. Substituted quinolines are historically among the most important antimalarial drugs and are expected to achieve a substantial reduction of malaria infections.

## Materials and methods

### Biogenesis of N1-(7-chloroquinoline -4-yl) ethane-1,2-diamine

A form of 4,7 dichloroquinoline (1.8 g, 0.01 mol) and ethylene diamine (0.06 g, 0.01 mol) was evaluated through thin layer chromatography (TLC) at the end of a chemical reaction. Filtration was used to remove the crystals of 4-substituted 7-chloroquinoline. After acetone treatment the end compound was recrystallized twice providing N1-(7-chloroquinoline-4-yl) ethane-1,2-diamine (CAS Number-5407-57-8).

### N1-(7-chloroquinoline-4-yl) ethane-1,2-diamine in silico analysis

The synthesized compound N1-(7-chloroquinoline-4-yl) ethane-1,2-diamine was analyzed for its cytotoxic potential using the Osiris protocol from its official website (https://www.organic-chemistry.org/prog/peo/). Parts of the Lipinski rule of five important parameters were utilized for quantification in order to trace their biological functions.

### *Anopheles stephen*s*i* and *Aedes aegypti* cultures

Developmental instars of *Anopheles stephensi* and *Aedes aegypti* eggs were maintained at the following conditions of the laboratory: 27 ± 2 °C, 75–85% R.H. and 14 h:10 h (L:D) photoperiod.

### Toxicity effects on developmental instars of *Aedes aegypti and A. stephensi*

The mosquitoes *A. aegypti* and *A. stephensi* were cultured and maintained following Murugan et al.^37^. For toxicology studies, 25 individuals of both *A. stephensi* and *A. aegypti* larva (1st, 2nd, 3rd, and 4th) and pupae were placed for a 24 h treatment in a tank filled with 500 mL of distilled water at concentrations of 4,7-dichloroquinoline (2, 4, 6, 8 and 10 ppm)^38^. In each treatment, 3 replications were carried out, in addition to negative controls. Mortality rate in percentage was studied applying the following formula:

$${\text{Mortality }}\left( \% \right) = \frac{{{\text{Number}}\,{\text{ of}}\,{\text{ dead}}\,{\text{ individuals}}}}{{{\text{Number}}\,{\text{ of}}\,{\text{ treated}}\,{\text{ individuals}}}} \times 100.$$

### Antiplasmodial cell culture assays on *P. falciparum*

CQ-sensitive strain 3D7 and CQ-resistant strain INDO of *Plasmodium falciparum* were used to test the antimalarial activity of 4,7-dichloroquinoline. They were maintained according to the method described by Murugan et al.^39^. Formulations of 4,7-dichloroquinoline in DMSO were evaluated by the procedure of Murugan et al.^40^, modified after Smilkstein et al.^41^. Microscopic examination of Giemsa stained smear samples of normal *Plasmodium falciparum* exposed to 4,7-dichloroquinoline was following Bagavan et al.^42^.

### In vivo antiplasmodial assays on *P. falciparum*

Following the method of Murugan et al.^43^, male albino mice (weight 27–30 g) were tested. They were maintained as reported by Murugan et al.^43^. For each experiment, three albino mice were used to test the antimalarial potential of the synthesized compound, 4,7-dichloroquinoline following a four-day inhibition technique by Murugan et al.^43^. Chloroquine (Sigma-Aldrich, Germany) was used as a positive control drug with normal saline (0.9%) at 5 mg/kg, while the negative control group was treated with 1 mL deionized water. The parasites inoculated in mice were noticed after 4 days of infection through microscopic observations of the blood^44^. Chemosuppression (%) was analyzed for every concentration of the parasitemia following the method of Argotte et al.^45^.

### Infection and toxicity towards cells

We procured Vero cells from the National Center for Cell Science (NCCS Maharashtra, India). The medium used for cultivation (EMEM) contained 10% fetal bovine serum and was incubated at 37 °C in a 5% CO_2_ atmosphere. We decreased the serum concentration to 2% when viral cultures were used. As described by Murugan et al.^43^ Dengue virus type-2 (DEN-2) New Guinea C strain was raised through adopting the cell line and were retrieved after the expression of cytopathic effects (CPE), commonly seven days after infection. Infected viral cells were stored at – 70 °C. Cytotoxicity assays and viral quantification assays were following Sujitha et al.^46^ with minor modification.

### Statistical analysis

Data from Probit analysis allowed the analysis of the effective lethal concentrations of the mosquito larvicidal and pupicidal experiments^47^. From the drug concentration–response curves the IC_50_s of *Plasmodium* were calculated. In vivo antimalarial data were checked for normality and analysed using ANOVA with two factors (i.e. dose and treatment). DEN-2 PFU and cytotoxicity data were determined by ANOVA followed by the HSD test of Tukey with the following probabilities (P = 0.05). All analyses were commonly carried out with the SPSS software package version 16.0.

## Results and discussion

### N1-(7-chloroquinoline-4-yl) ethane-1, 2-diamine effects analyzed by in-silico approaches

The synthesized compound showed no tumorigenic, irritative, nor reproductively significant effects in silico. Besides, LogP and LogS values (Table 1) indicated that the synthesized compound was hydrophilic with a high probability of being distributed along with hydrophilic environments such as insect lymph or cellular cytosol. Molinspiration analysis indicated that the values regarding, GPCR ligand, kinase inhibitor, nuclear receptor ligand, ion channel modulator, protease inhibitor and enzyme inhibitor scores were high. Molinspiration analysis generally indicated that the larger the value of the score was, the more the compound would have biological effects. Therefore, according to in silico analysis, N1-(7-chloroquinoline-4-yl) ethane-1,2-diamine is likely to affect ion channels, kinases, and some important enzymes. The above results could be related to acute toxicity on young instars of *A. aegypti* and *A. stephensi* and highly increased the growth inhibition of *Plasmodium falciparum*^48^. The in silico study highlighted that quinoline derivatives (BT24) effectively inhibited all four dengue serotypes (1–4) of infected Vero cells by compound (BT24) binding to the active site of the DENV-2 protease. On the other hand, no cytotoxic in silico results could be corroborated by the effect of Vero cell line studies. The drug likeness value is similar to quinolineb (− 1.65, data not shown) as the compounds are closely related. As a result, the compound could be used for the above mentioned applications.

**Table 1:** Informatic analysis results using Osiris (https://www.organic-chemistry.org/prog/peo/) and Molinspiration (https://www.molinspiration.com, Slovensky Grob, Slovakia) software.

Properties	Compound
	*N*^1^-(7-chloro quinoline -4-yl) ethane-1,2-diamine
Molecular weight (g/mol)	221
LogP	1.31
LogS	− 2.99
TPSA	50.94
GPCR ligand	− 0.01
Ion channel modulator	0.42
Kinase inhibitor	0.41
Nuclear receptor ligand	− 0.92
Protease inhibitor	− 0.22
Enzyme inhibitor	0.24
Mutagenic	3
Tumorigenic	1
Irritant	1
Reproductive effect	1
Druglikeness	− 1.35

The toxicity risk is expressed considering the following number code: (1) no risk (2) medium risk (3) high risk.

### Toxicity effect of 4,7-dichloroquinoline on *A. aegypti* and *A. stephensi*

In agreement with the current research, Saini et al.^49^ studied the antimalarial potential of quinoline-pyrazolo pyridine derivatives. Mosquitocidal results revealed that the synthesized 4,7-dichloroquinoline was highly toxic to developmental stages of malarial and dengue vectors providing LC_50_ values ranging from 4.408 µM/mL (larva I) to 7.958 µM/mL (pupa) for the chosen malaria vector and 5.016 µM/mL (larva I) to 10.669 µM/mL (pupa) for the dengue vector (Table 2). Recently, Rueda et al.^50^ demonstrated both adulticidal and larvicidal activity of *A. aegypti* when exposed to synthesized α-amino nitriles. Shao et al.^51^ showed for hexahydroimidazo [1,2-α] pyridine derivatives that they had excellent pesticidal properties against aphid species. Furthermore, Sun et al.^52^ highlighted that piperazinedione derivatives were highly toxic on the root-knot nematode *Meloidogyne incognita*. The K1 strain being resistant against chloroquine (CQ) was shown by Gayam and Ravi^53^ and that cinnamoylated chloroquine hybrid analogues showed highest antimalarial activity. Lastly, Kondaparia et al.^54^ found that 4-aminoquinolines showed considerable antimalarial activity on *Plasmodium falciparum*. It was proposed that death rate caused by 4,7-dichloroquinoline for the different life stages of larval populations of both *A. stephensi* and *A. aegypti* may be due to the upregulation of electronegative ions which provided better biological activity on target pests^55^. Indeed, Rahuman et al.^56^ reported that *Zingiber officinale* derived molecules showed toxicity on the 4th larval stages of the dengue vectors belonging to *Culex* species.

**Table 2:** Acute toxicity of synthesized 4,7-dichloroquinoline on young instars of *Anopheles stephensi* and *Aedes aegypti.*

Species	Target	LC_50_ (LC_90_) (µM/mL)	95% Confidence Limit LC_50_ (LC_90_) µM/mL		Regression equation	χ^2^ (*df* = 4)
			Lower	Upper		
*Anopheles stephensi*	Larva I	4.408 (8.145)	3.001 (6.819)	5.487 (11.046)	*y* = 1.512 + 0.343*x*	8.699 *n.s*
	Larva II	4.916 (9.160)	4.469 (8.507)	5.333 (10.021)	*y* = 1.484 + 0.302*x*	4.132 *n.s*
	Larva III	5.572 (10.562)	5.084 (9.726)	6.043 (11.707)	*y* = 1.431 + 0.257*x*	0.924 *n.s*
	Larva IV	6.304 (12.102)	5.767 (10.994)	6.858 (13.699)	*y* = 1.393 + 0.221*x*	1.586 *n.s*
	Pupa	7.958 (15.159)	7.264 (13.347)	8.849 (18.060)	*y* = 1.416 + 0.178*x*	2.832 *n.s*
*Aedes aegypti*	Larva I	5.016 (12.451)	4.243 (11.018)	5.684 (14.725)	*y* = 0.864 + 0.172*x*	0.554 *n.s*
	Larva II	5.998 (14.198)	5.244 (12.363)	6.753(17.265)	*y* = 0.938 + 0.156*x*	0.890 *n.s*
	Larva III	7.838 (17.484)	6.949 (14.712)	9.074 (22.646)	*y* = 1.041 + 0.133*x*	0.736 *n.s*
	Larva IV	9.505 (19.900)	8.339 (16.360)	11.504 (26.984)	*y* = 1.172 + 0.123*x*	1.691 *n.s*
	Pupa	10.669 (20.355)	9.353 (16.818)	13.013 (27.294)	*y* = 1.412 + 0.132*x*	0.710 *n.s*

*Control* no mortality, *LC*_*50*_ lethal concentration that kills 50% of the exposed organisms, *LC*_*90*_ lethal concentration that kills 90% of the exposed organisms, *χ*^*2*^ chi-square value, *d.f.* degrees of freedom, *χ*^*2*^ 0.05 level of significance indicates homogeneity of results.

### Antiplasmodial activities

**Figure 2:**
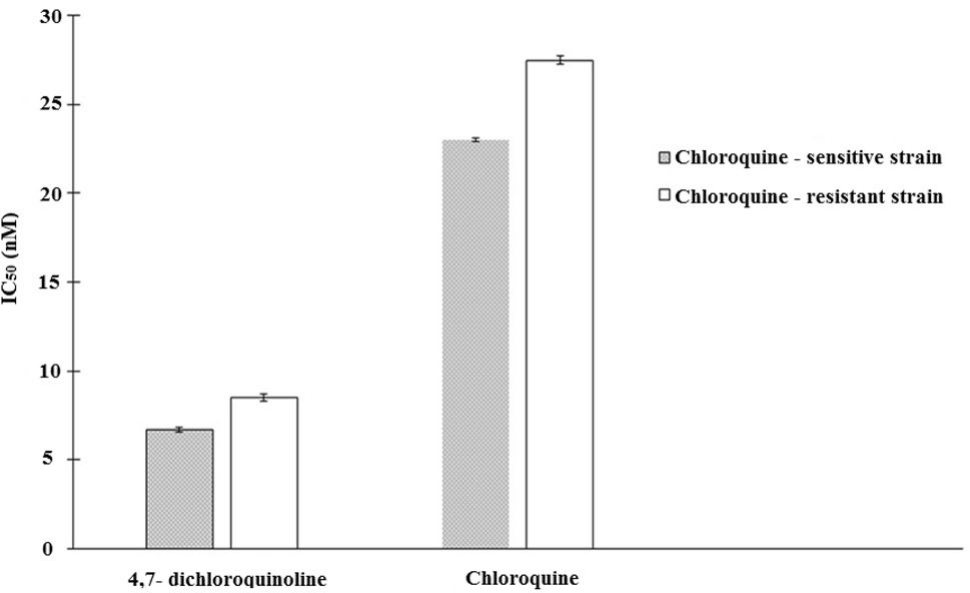
In vitro growth inhibition of chloroquine-sensitive and chloroquine-resistant strains of *Plasmodium falciparum* post-treatment with 4,7-dichloroquinoline and chloroquine. T-bars represent standard deviations.

As a result of antiplasmodial assays, when compared to chloroquine, the synthesized 4,7-dichloroquinoline expressed significant growth inhibition against both CQ-resistant (CQ-r) and CQ-sensitive (CQ-s) strains of *P. falciparum* (Fig. 2). Similarly, Kumawat et al.^57^ investigated 7-Chloro-4-aminoquinoline derivatives causing moderate growth inhibition on CQ-sensitive *P. falciparum* (RKL-2). Also, Faruk Khan^58^ noticed that the cyclen 4-Aminoquinoline anlog, bisquinoline, exhibited in vitro and in vivo antiplasmodial properties on D6 W2 chloroquine-sensitive and chloroquine-resistant strains of *P. falciparum* with IC_50_ values of 7.5 nM (D6 CQ-sensitive) and 19.2 nM (W2 CQ-resistance). Very recently, Pinheiro et al.^59^ showed that quinoline and non-quinoline derivatives were highly effective against both *P. falciparum* W2 chloroquine-resistant strains of *P. falciparum* in infected mice. Quinoline drugs exhibited potential inhibitory effect of proteolysis, DNA replication, RNA synthesis and heme polymerization in *Plasmodium* spp^60,61^. Additionally, Aboelnaga and El-Sayed^62^ reported that 7-chloroquinoline derivatives showed significant anticancer activity on cervical (Hela) cancer cell lines, human breast cancer (MCF-7) and colon carcinoma (HCT-116). Protein kinase inhibitors, topo isomerase inhibitors, carbonic anhydrase inhibitors, Hsp90 inhibitors are the anticancer mechanisms of quinoline derivatives^63^. Aderibigbe et al.^64^ found that polymer loaded aminoquinoline were highly potent against the strain of *P. falciparum* which was chloroquine-sensitive. A new quinoline derivative, thiazolyl hydrazone were synthesized as effective antifungal and anticancer agents by Erguc et al.^65^.

**Figure 3:**
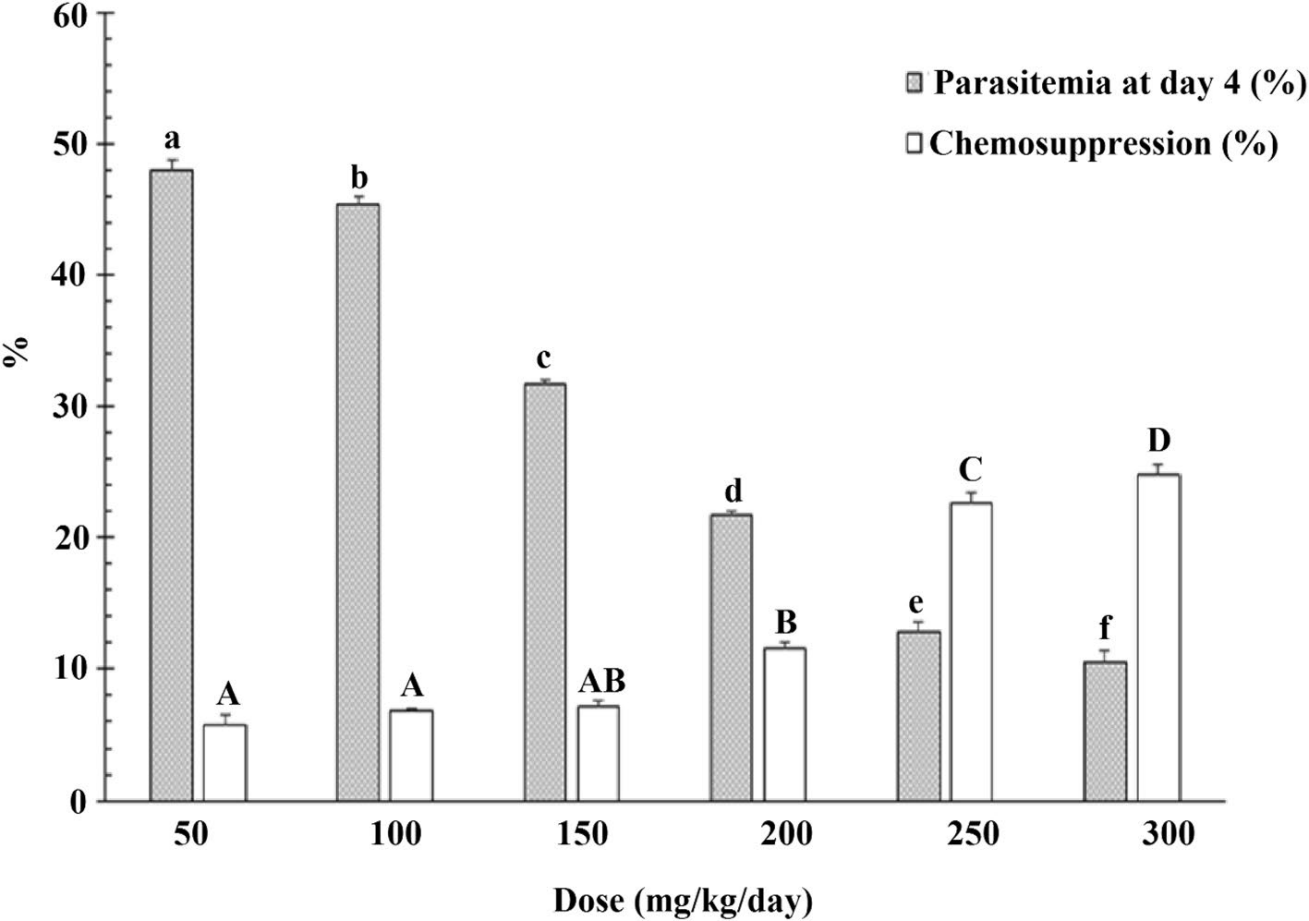
In vivo growth inhibition of *Plasmodium falciparum* parasites infecting albino mice post-treatment with 4,7-dichloroquinoline. Positive control (chloroquine 5 mg/kg/day) led to mean parasitemia of 1.0 ± 0.0% at day 4. T-bars represent standard deviations. Above each column, different letters indicate significant differences (ANOVA, Tukey's HSD, *P* < 0.05).

Dose-dependent chemosuppression against *P. falciparum* was demonstrated by Peters’ 4-day chemo-suppressive activity assay (Fig. 3). After 4 days of 4,7-dichloroquinoline treated groups exhibited the percentage of parasitemia 10.6 ± 0.8% at 300 mg/kg/day than that of the control drug chloroquine (CQ) 1.0 ± 0.0%^37,66^. Tang et al.^67^ showed antimalarial activities against the *P. falciparum* strain K173 with EC_50_ values ranging from 0.38 to 0.43 mg/kg. Manohar et al.^68^ found that 4-Aminoquinoline-pyrimidine hybrids exhibited 80% parasitemia suppression as compared to CQ (20%). Finally, Sahu et al.^69^ found that low doses of tigecycline (3.7 mg/kg) showed 77–91% of parasitaemia suppression. Inhibition of parasitaemia of 77–91% was provided by 3.7 mg/kg dose of tigecycline for 4 consecutive days. Furthermore, the authors reported that in vivo treatment with tigecycline in combination with sub-curative doses of CQ provided 100% mortality of *P. falciparum* in infected mice.

### Cytotoxicity effect of 4,7-dichloroquinoline on Vero cells

**Figure 4:**
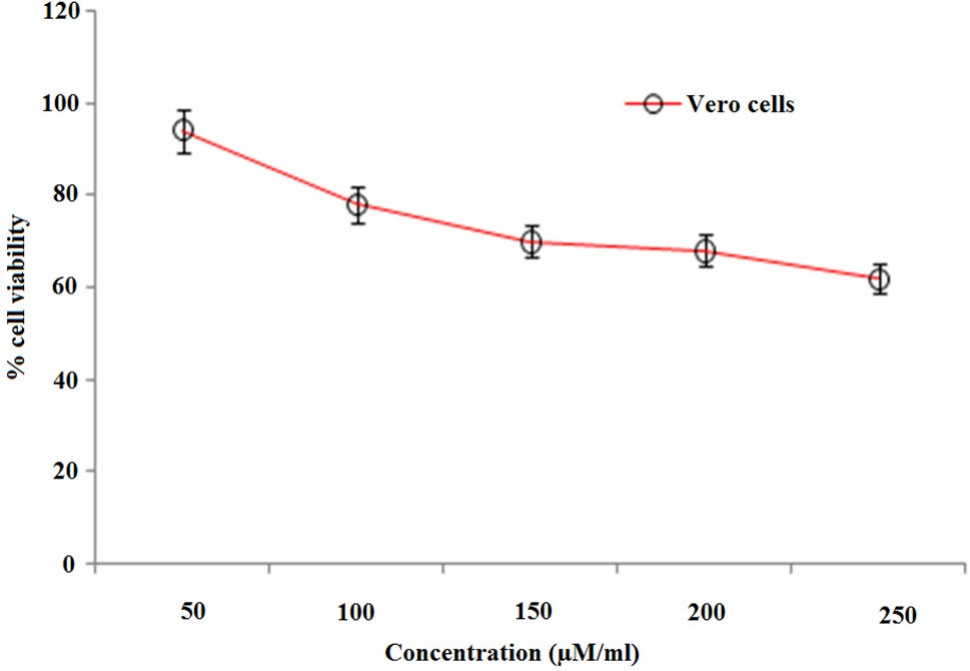
Cytotoxic effects of 4,7-dichloroquinoline on Vero cells.

**Figure 5:**
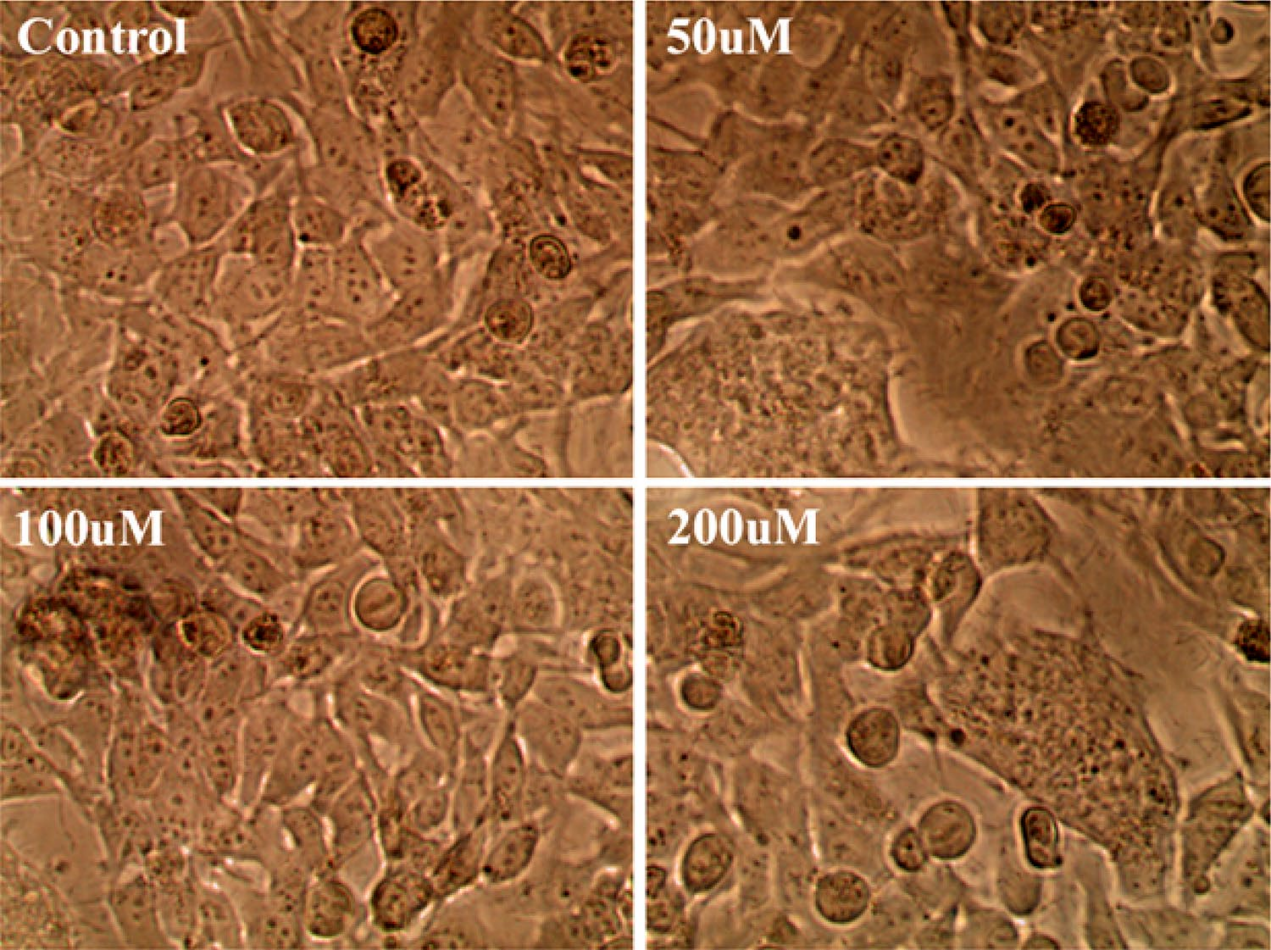
Vero cell viability after the treatment with different concentrations of 4,7-dichloroquinoline.

In the present study, the viability of Vero cells was incorporated in various concentrations of 4,7-dichloroquinoline^70^. We observed that there were no adverse morphological differences in the treated groups when compared to control Vero cells (Figs. 4, 5). For example, Tseng et al.^71^ studied that the new derivatives of synthesized 2-aroyl-3-arylquinoline compounds provided substantial cytotoxicity against Huh-7 cells with less than 20% viability at doses of 100 μM of 4,7-dichloroquinoline. Cell death above a concentration of 60 μM of 4-methyl pyrimido (5,4-c) quinoline-2,5(1H, 6H)-dione on MDCK cells were shown by Paulpandi et al.^34^. Recently, Beesetti et al.^72^ highlighted that quinoline derivatives, BT24 effectively inhibit DENV-2 protease with IC_50_ of 0.5 μM.

### Antiviral effects of 4,7-dichloroquinoline on dengue and zika virus

**Figure 6:**
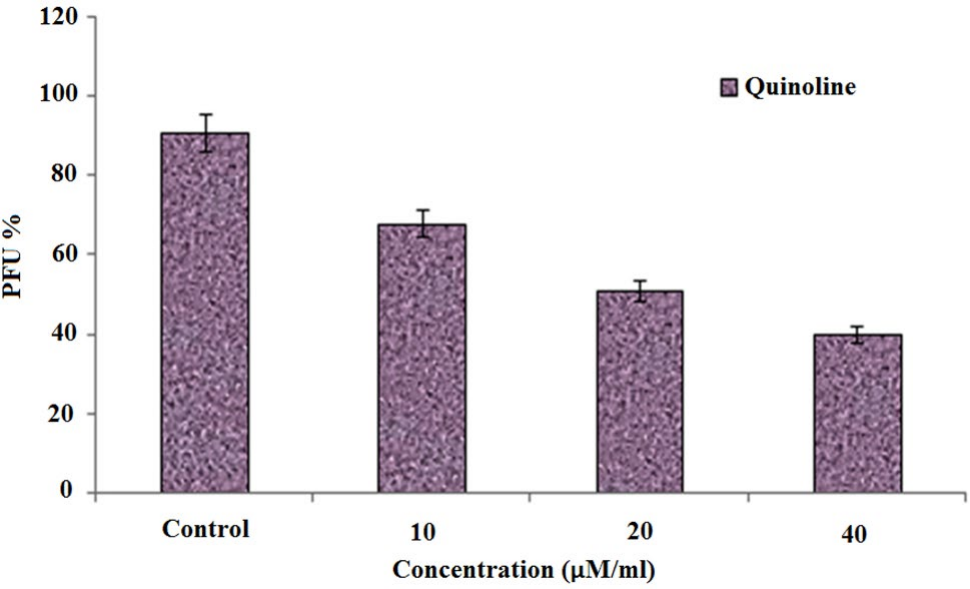
Inhibition of dengue virus (DEN-2) post-treatment with 4,7-dichloroquinoline.

**Figure 7:**
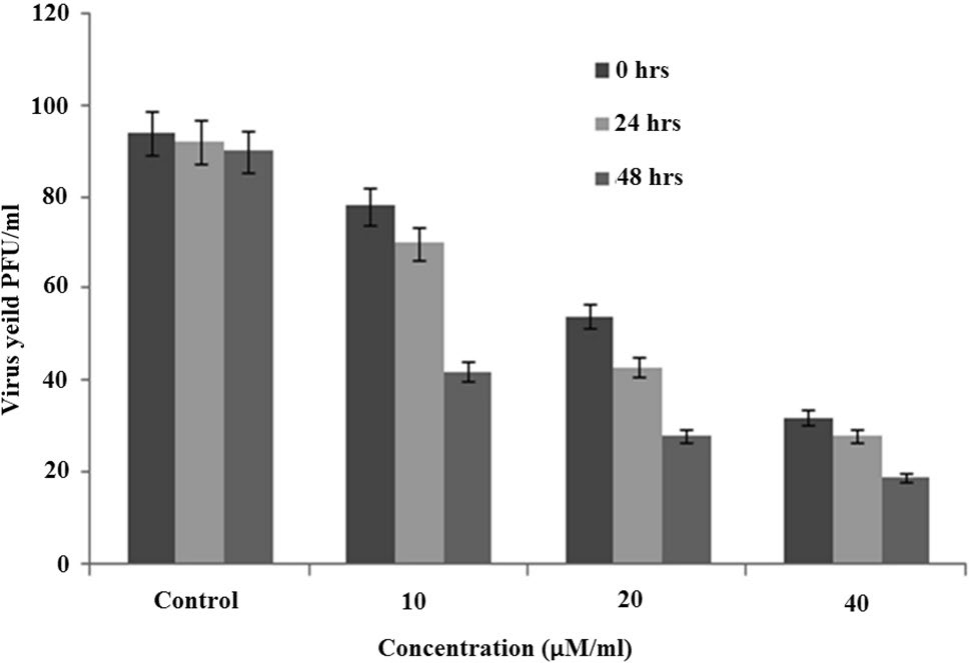
Post treatment reduction in DEN-2 viral yield with 4,7-dichloroquinoline at different time intervals.

Antiviral results showed that the synthesized compound, 4,7-dichloroquinoline tested at 10–40 μg/mL significantly inhibited dengue virus (DENV-2), with a reduction of PFU abundance^73^ (see also Fig. 6). Furthermore, a plaque assay displayed after an individual exposure and with a minimum dosis that 4,7-dichloroquinoline effectively inhibited the production of dengue viruses. Post 48 h treatment duration of the viral production was 91 PFU/mL in the control, whereas it was 19 PFU/mL, after the treatment of in 4,7-dichloroquinoline at a concentration of 40 μL/mL (Fig. 7). Similarly, Guardia et al.^74^ discovered that quinoline derivatives highly inhibited DENV-2 with IC_50_ values ranging from 3.03 to 0.49 μM, respectively. Very recently, Devaux et al.^75^ found that chloroquine/hydroxychloroquine significantly inhibited pandemic SARS-CoV-2. Furthermore, chloroquine highly inhibited HCoV-229E replication in epithelial lung cell cultures^76^. It became apparent that the Zika virus provided a regional threat for Latin America and the Caribbean^77,78^.

## Conclusion

It is clear from previous reports that resistance to the malaria vector continues to grow. This is increasingly limiting our ability to control malaria worldwide. Our present study demonstrated the mosquitocidal potential of 4,7-dichloroquinoline derivatives against the key mosquito vectors, *An. stephensi* and *Ae. aegypti*. This would be a promising advance in the development of clean, non-toxic, and environmentally acceptable quinoline compounds for their effect against mosquito vectors. Furthermore, 4,7-dichloroquinoline had a significant and promising anti-malarial potential to reduce the global threat malaria. Quinoline decreased virus proliferation and replication during protein synthesis at mRNA levels. No cell cytotoxicity was identified. A compound was recognized as a unique kind of structure different for additional improvement against DENV specialists. We have presented here novel quinoline subordinates that are fundamentally dynamic against dengue infection in a partially subordinate way. The discoveries presented here are significant as a starting point for additional clarification of the particular components of the antiviral action and to pick up the necessary information to additionally grow new, compelling, strong, and safe medications to lessen the risks from viral diseases.

## Supplementary Information

Supplementary Information.
