# Supplementary material for: Synthesis of new series of quinoline derivatives with insecticidal effects on larval vectors of malaria and dengue diseases
Source: Sci Rep. 2022 Mar 19;12:4765. doi: 10.1038/s41598-022-08397-5 (PMC8933857; doi:10.1038/s41598-022-08397-5)

**Infrared spectral data: (KBr, υ in cm^-1^)**: 639.91 (w), 703.19 (w), 750.55 (s), 817.85 (s), 850.76 (w), 878.58 (s), 945.78 (w), 986.46 (s), 1074.93 (w), 1110.50 (s), 1204.99 (w), 1246.46 (s), 1327.56 (s), 1417.83 (s), 1455.94 (w), 1497.04 (s), 1574.75 (s), 1605.06 (w), 1930.02 (w), 1994.03 (w), 2088.53 (w), 2207.47 (w), 2315.68 (w), 2448.22 (w), 3071.56 (w), 3113.86 (w), 3254.53 (w), 3373.42 (s)

**^1^H NMR spectra: (400 MHz, d_6_- DMSO); - value in ppm**: 8.88 (s, 1H, Ar-N*H*); 8.24-8.18 (m, 2H, Ar-*H*); 7.91 (d, J=6.8Hz, 1H, Ar-*H*); 7.64 (s, 1H, Ar-*H*); 7.16 (d, J=7.2Hz, 1H, Ar-*H*); 3.81 (t, J=7.0Hz, 2H, Ar-N-C*H*_2_); 2.64 (t, J=6.8Hz, 2H, NH_2_-C*H*_2_)

**Infrared spectral data: (KBr, υ in cm^-1^)**: 627.43 (w), 707.75 (s), 813.21 (s), 851.42 (w), 978.82 (w), 1015.71 (w), 1078.07 9w), 1109.65 (s), 1186.31 (w), 1309.78 (s), 1406.52 (m), 1511.86 (w), 1309.78 (s), 1406.52 (w), 1511.86 (s), 1709.70 (w), 1929.48 (w), 2099.31 (w), 2056.13 (w), 2215.41 (w), 2320.47 (w), 3328.61 (s).

**^1^H NMR spectra: (400 MHz, d_6_- DMSO); - value in ppm**: 8.89 (s, 1H, Ar-N*H*); 8.25-8.19 (m, 2H, Ar-*H*); 7.91 (d, J=7.0Hz, 1H, Ar-*H*); 7.65 (s, 1H, Ar-*H*); 7.17 (d, J=7.0Hz, 1H, Ar-*H*); 3.87 (t, J=6.8Hz, 2H, Ar-N-C*H*_2_); 2.62 (t, J=7.2Hz, 2H, NH_2_-C*H*_2_); 1.41 (m, 4H, CH_2_-(C*H*_2_)_2_-CH_2_)

**Infrared spectral data: (KBr, υ in cm^-1^)**: 627.46 (w), 681.98 (m), 708.26 (s), 748.58 (w), 812.07 (s), 849.34 (w), 976.64 (m), 1015.93 (m), 1076.12 (m), 1109.56 (s), 1183.47 (s), 1236.24 (s), 1323.2 (s), 1405.88 (m), 1508.78 (s) 1538.80 (w), 1712.01 (w), 1927.61 (w), 1992.87 (w), 2050.60 (w), 2100.19 (w), 2853.49 (w), 2923.98 (w), 3081.83 (w), 3340.71 (w).

**^1^H NMR spectra: (400 MHz, d_6_- DMSO); - value in ppm**: 8.89 (s, 1H, Ar-N*H*); 8.24-8.19 (m, 2H, Ar-*H*); 7.92 (d, J=7.2Hz, 1H, Ar-*H*); 7.64 (s, 1H, Ar-*H*); 7.21 (d, J=7.4Hz, 1H, Ar-*H*); 3.88 (t, J=7.2Hz, 2H, Ar-N-C*H*_2_); 2.62 (t, J=7.0Hz, 2H, NH_2_-C*H*_2_); 1.42 (m, 4H, CH_2_-C*H*_2_-(CH_2_)_2_-C*H*_2_-CH_2_); 1.03 (m, 4H, (CH_2_)_2_-(C*H*_2_)_2_-(CH_2_)_2_)

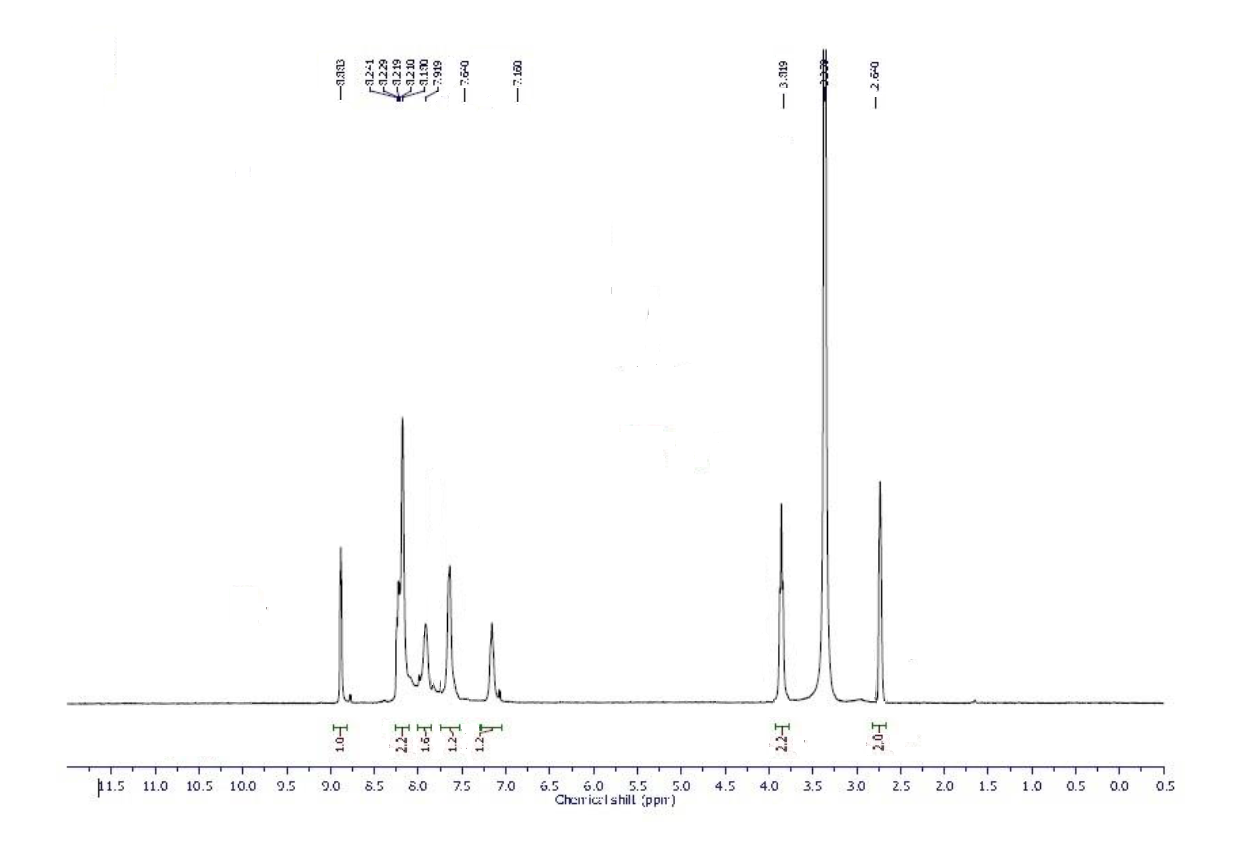

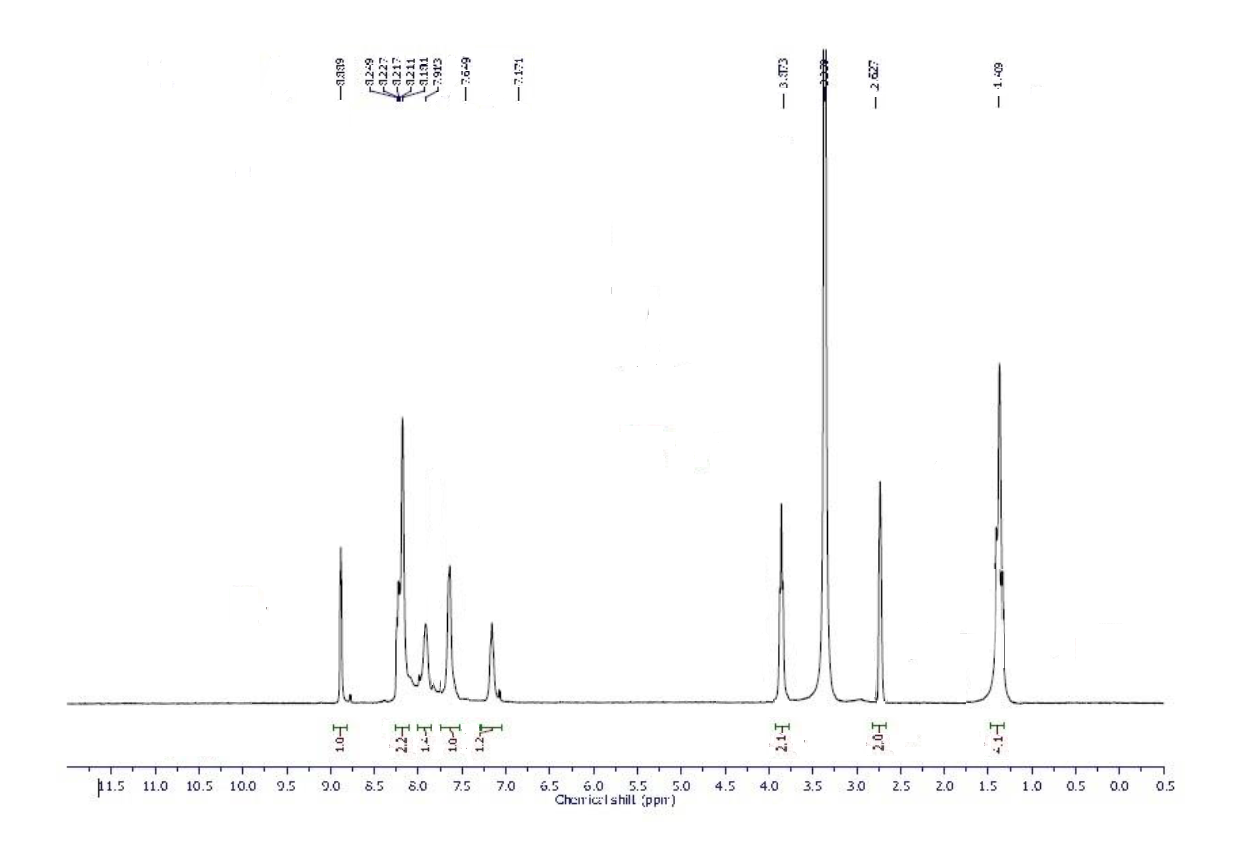

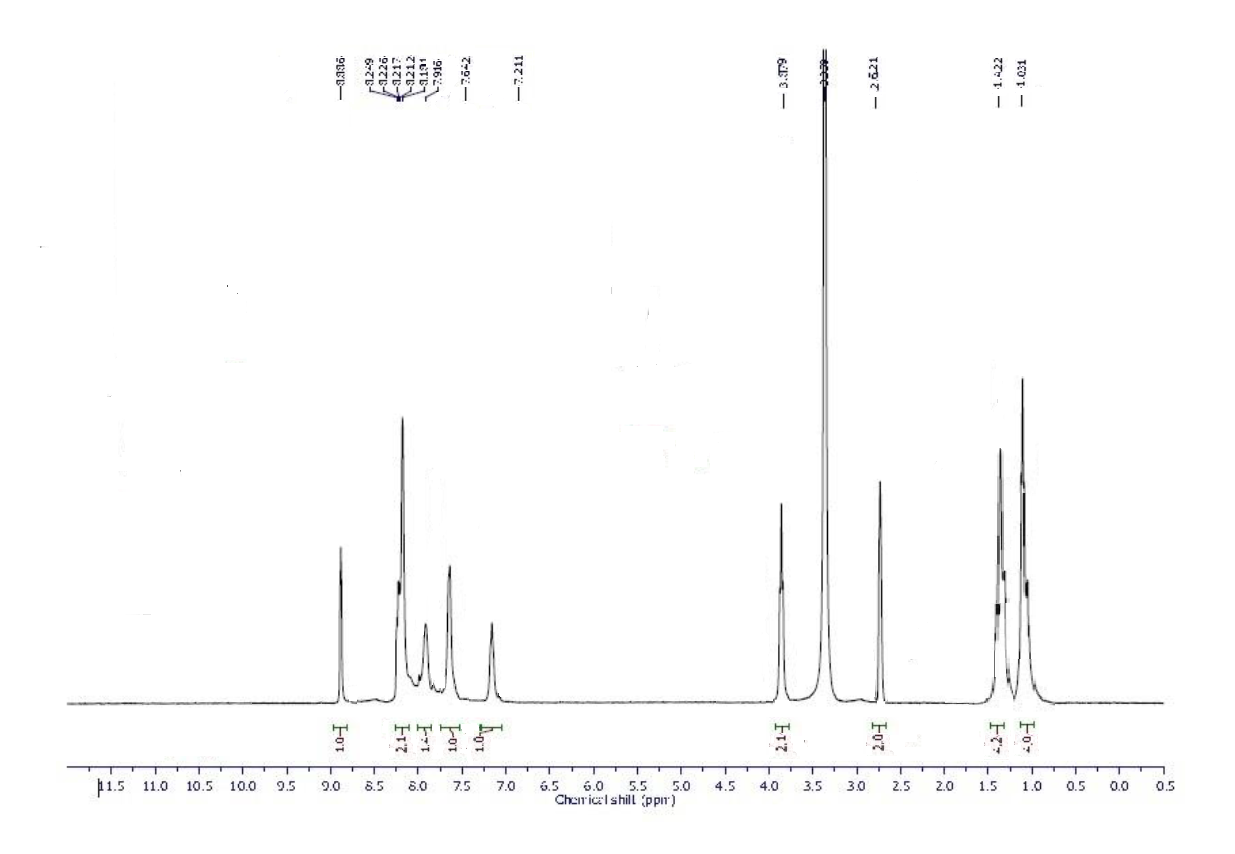

Supplement: Supplementary file 1 — Supplementary Information. [file 41598_2022_8397_MOESM1_ESM.docx]
